# Establishing and Governing e-Mental Health Care in Australia: A Systematic Review of Challenges and A Call For Policy-Focussed Research

**DOI:** 10.2196/jmir.4827

**Published:** 2016-01-13

**Authors:** Carla Meurk, Janni Leung, Wayne Hall, Brian W Head, Harvey Whiteford

**Affiliations:** ^1^School of Public HealthFaculty of Medicine and Biomedical SciencesThe University of QueenslandHerstonAustralia; ^2^Centre for Youth Substance Abuse ResearchThe University of QueenslandHerstonAustralia; ^3^Institute of Social Science ResearchThe University of QueenslandSt LuciaAustralia; ^4^Policy and Epidemiology GroupQueensland Centre for Mental Health ResearchArcherfieldAustralia

**Keywords:** telemedicine, e-health, e-mental health, e-therapy, Internet, online, cognitive behavioural therapy, anxiety, anxiety disorders, depression, depressive disorder, Australia, research translation, evidence-informed policy, implementation

## Abstract

**Background:**

Growing evidence attests to the efficacy of e-mental health services. There is less evidence on how to facilitate the safe, effective, and sustainable implementation of these services.

**Objective:**

We conducted a systematic review on e-mental health service use for depressive and anxiety disorders to inform policy development and identify policy-relevant gaps in the evidence base.

**Methods:**

Following the PRISMA protocol, we identified research (1) conducted in Australia, (2) on e-mental health services, (3) for depressive or anxiety disorders, and (4) on e-mental health usage, such as barriers and facilitators to use. Databases searched included Cochrane, PubMed, PsycINFO, CINAHL, Embase, ProQuest Social Science, and Google Scholar. Sources were assessed according to area and level of policy relevance.

**Results:**

The search yielded 1081 studies; 30 studies were included for analysis. Most reported on self-selected samples and samples of online help-seekers. Studies indicate that e-mental health services are predominantly used by females, and those who are more educated and socioeconomically advantaged. Ethnicity was infrequently reported on. Studies examining consumer preferences found a preference for face-to-face therapy over e-therapies, but not an aversion to e-therapy. Content relevant to governance was predominantly related to the organizational dimensions of e-mental health services, followed by implications for community education. Financing and payment for e-services and governance of the information communication technology were least commonly discussed.

**Conclusions:**

Little research focuses explicitly on policy development and implementation planning; most research provides an e-services perspective. Research is needed to provide community and policy-maker perspectives. General population studies of prospective treatment seekers that include ethnicity and socioeconomic status and quantify relative preferences for all treatment modalities are necessary.

## Introduction

Growing evidence attests to the efficacy of Internet-assisted therapeutic tools, particularly in the treatment of common mental disorders such as mild to moderate depressive and anxiety disorders [1]. Prominent mental health researchers and advocates are optimistic about the potential for e-mental health care to enhance accessibility and increase cost efficiency of services, promote consumer empowerment, and overcome geographic obstacles to service utilization [2-6]. They have called on government to support and promote the development and implementation of these tools [7,8]. Recent translational research has detailed the organizational infrastructure that exists in Australia and called for further translational research focused on closing the evidence-practice gap, ensuring the viability of e-services through financing and enhancing the reach of, and adherence to, e-therapies especially through health promotion [7,9].

Realizing the potential of these technologies, however, will require that these treatments are embedded within the existing health system as part of a continuum of mental health care and alongside other modalities such as face-to-face psychological treatment and pharmacotherapies.

While meta-analyses show that Internet-based and Internet-assisted therapies are effective and have an important role in the Australian health system, evidence that these interventions *can* work under experimental conditions is not sufficient to show that an intervention *should* be upscaled and implemented from economic, social, and ethical perspectives [10,11]. Nor does it precisely describe how these services will operate within a health system [12-14]. More to the point, evidence on what works in achieving positive treatment outcomes in controlled trials does not necessarily provide information about *how* health policy makers and health professionals might act to implement these new technologies at scale using conventional policy mechanisms and changing established clinical practices [13].

**Figure 1:**
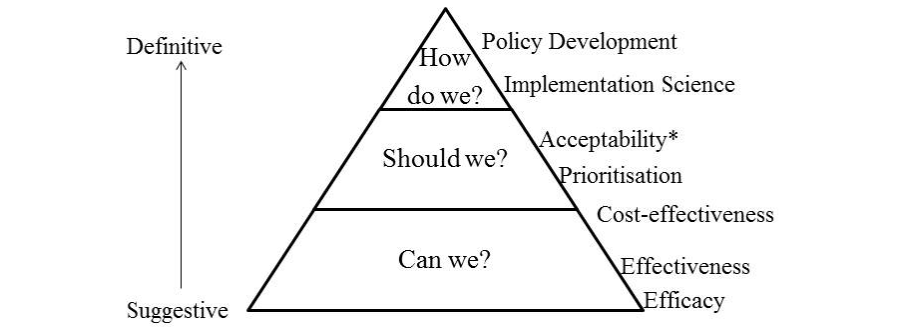
Hierarchy of policy-relevant information.

We can think about the informational requirements for introducing a new technology into the health care system in terms of a hierarchy of policy-relevant information (see Figure 1). This is a hierarchy based on information type rather than methodological rigor. Under this view, efficacy and effectiveness studies—randomized controlled trials, systematic reviews, and meta-analyses—which constitute the pinnacle of a hierarchy of evidence types within the biomedical sciences, form the bedrock for subsequent investigations of the cost-effectiveness, acceptability, and logistics of implementing efficacious technologies. The hierarchy of policy relevance is not immutable, and stages of development are interrelated to some degree. For example, the acceptability of technologies can be optimized through incorporating user preferences into the development of technologies as well as through promotion of fully developed ones.

These informational requirements apply, in different ways, to multiple domains: clinical settings, research settings, communities, and within government. Achieving successful implementation depends on harmonizing interacting processes that are initiated in each domain. Thus, a pluralist approach needs to be taken as to what constitutes relevant and useful information to facilitate implementation in different contexts. Clinician, research, community, and policy-maker perspectives all need to be carefully enumerated to ascertain how particular issues are framed, identify mechanisms for action, and describe the scope and limits of what can feasibly and ethically be changed in, and through, each domain in order to facilitate uptake.

The objective of this systematic review was to take stock of what is currently known about the utilization of e-mental health, interpreted from a policy-making perspective on implementation. Our aims were to (1) identify current knowledge about e-mental health service utilization in Australia for depressive and anxiety disorders, (2) synthesize evidence relevant to e-mental health policy development, and (3) identify future directions for policy-focused research.

## Methods

This systematic review employed an a priori protocol based on the Preferred Reporting Items for Systematic Reviews and Meta-Analyses Protocol (PRISMA-P) 2015 guidelines [15]. The research questions and inclusion criteria were established before the review started through meetings, written proposals, and discussions between the authors. CM and JL were responsible for identifying and reviewing literature. Disagreements in screening and data extraction were resolved through consensus meetings between CM and JL. Data were stored in Endnote and Excel. Sources were appraised according to their study design, using standard quality criteria. Data were synthesized by area and level of policy relevance. We report the results in accordance with the PRISMA statement [16].

### Eligibility Criteria

The research question and eligibility criteria were formulated based on a PICO model (Population, Intervention or factors, Comparison, and Outcome). The population of interest was Australia. Literature from New Zealand, United Kingdom, Norway, Sweden, Finland, and Canada were included in the initial search in case an insufficient number of studies were found in Australia. However, sufficient Australian studies were found, and thus international studies were excluded at the screening stage. Our outcome of interest was the use of e-mental health services for depressive (affective) disorders or anxiety disorders (International Classification of Diseases, 10^th^ revision, codes F30 to F44). E-mental health is a relatively new and rapidly evolving field. Thus, only published literature and gray literature from 2005 were included.

### Search and Study Selection

The search was conducted during February 2015. A research librarian was consulted regarding the search strategy. Limits used were English language, human subjects, and dates from 2005-2015. Gray literature and peer-reviewed publications were included in our search. Databases searched included Cochrane, PubMed, PsycINFO, CINAHL, Embase, ProQuest Social Science, and Google Scholar. We reviewed the references in the final included studies to find additional research studies, as part of our supplementary search.

Search terms used were MeSH (Medical Subject Headings) for Cochrane and PubMed, Thesaurus of Psychological Index Terms for PsycINFO, CINAHL headings for CINAHL, EMTREE for Embase, ProQuest Social Science, and Google Scholar. For each of these databases, the general search strategy took the following form: 

(((e-health & (affective disorder OR anxiety disorder)) OR e-mental health) & (<list of factors associated with use, eg service use OR barriers OR attitude OR socio-economic OR preference>)). Using PubMed as an example, the search strategy was ((((Telemedicine) OR Therapy, Computer-Assisted)) AND ((((((((mental health) OR mood disorders) OR depression) OR anxiety disorders) OR anxiety) OR psychotherapy) OR mental health service) OR community mental health services))) AND (((((((((Australia) OR New Zealand) OR United Kingdom) OR UK) OR Norway) OR Sweden) OR Finland) OR Canada)) AND ((((((((((((((Epidemiological factors) OR Health services accessibility) OR Health care disparities) OR Attitude to health) OR Health services research) OR Socioeconomic Factors) OR Demography) OR Social determinants of health) OR Health literacy) OR Patient satisfaction)) OR (Prefer* OR Challeng* OR Barrier* OR Facilitat*))

From the search, all studies were compiled and duplicates were removed. The titles and abstracts of the studies were screened to remove irrelevant studies. The full texts of the studies were then screened by JL and CM on the eligibility criteria for inclusion in the systematic review.

### Data Extraction

Data extraction was conducted by JL and CM who compared the extracted data to ensure consistency in data collection methods. Study characteristics extracted included the study aims and information on the sample. The factors of interest included any variables that could be a facilitator or barrier for e-mental health service usage for help-seekers, for example, knowledge and attitudes, sociodemographic, psychological, technological, and environmental factors. We were also interested in institutional and organizational factors that might facilitate or impede the use of e-mental health via service provision. Finally, we were interested in assessing the character of studies conducted in this area, including study design and methods of analysis.

### Quality and Bias Assessment

This review differs from the usual aims of systematic reviews in the biomedical sciences in that we wished to analyze past studies in terms of how they might be used to inform government policy. Thus, while we appraised study quality, policy relevance was our key concern. In line with standard protocols, we undertook a quality assessment based on the levels of evidence of the National Health and Medical Research Council (NHMRC) Evidence Hierarchy [17]. Level I evidence included systematic reviews. There was no existing Level I evidence on this topic (ie, on e-mental health service use as opposed to systematic reviews on efficacy of e-therapies, of which there are several [1,18,19]). Level II evidence included randomized controlled trials, observational studies, or case-control studies. Level III evidence included qualitative interviews or focus groups, and Level IV included commentaries and expert opinions. Category IV articles were excluded from evaluation in our findings, as they did not present new empirical evidence. They were instead reviewed and referred to where relevant in our introduction and discussion.

The distinction between studies on samples based on service users and/or online help-seekers versus samples of prospective service users is an important consideration for this review and therefore the sample source was incorporated into the assessment criteria. Level of evidence ratings were labeled “EU” for studies on existing e-mental health service users and/or self-selected e-mental health help-seekers, “PU” for studies drawn from community/general population samples including prospective users, and “SP” for studies that sampled service providers.

### Synthesis of Results

Data were synthesized for analysis according to their level of policy relevance and area of policy relevance. These categories are defined below.

#### Levels of Policy Relevance

We assessed papers on a 3-point scale (Low, Mod, High) of policy relevance. Our intention was to qualitatively score items against the hierarchy of policy-relevant evidence shown in Figure 1. Policy relevance=“Low” were studies focused on showing that a treatment or intervention is clinically effective. Policy relevance=“Mod” were studies that justified implementation of an intervention and defined the parameters for an intervention’s usefulness. These include cost-effectiveness and prioritization studies, as well as analyses of the ethical and social acceptability of the broad-scale implementation of a particular treatment. Acceptability, from a policy perspective, has a different meaning to the way it is commonly used in clinical trials—although there is overlap. In clinical trials, acceptability refers to elements such as satisfaction with treatment and treatment compliance. For the purposes of this review, acceptability refers to the “attractiveness” and appeal of an intervention among a significant sector of society. Acceptability includes both a disposition to use an intervention oneself and support for the idea of the intervention, for example, that key sectors of the public believe that e-mental health is a good idea and that it is appropriate for the government to deliver some mental health services in this way. Policy relevance=“High” refers to studies that provide explicit, empirical, or analytical evidence to support particular approaches to facilitating and governing the delivery of e-mental health care.

#### Area of Policy Relevance

For each study, we identified how it contributed to an area of policy relevance. We labeled these Target Demographic (T), Facilitating Uptake (F), and Governing Mechanisms (G). These areas are not independent from one another, and each study could potentially contribute to more than one area of policy relevance.

Target Demographic (T) refers to findings relevant to understanding e-mental health service use among specific sectors of the population. To examine this aspect of the literature, we analyzed information about sample characteristics, study inclusion and exclusion criteria, means of sample recruitment, mental health disorder targeted, phase of intervention (prevention or treatment), and platform or mode of e-mental health service.

Facilitating Uptake (F) refers to findings that are useful in understanding what characteristics explain willingness to use e-mental health care and under what conditions e-mental health will be attractive to different groups of people. To examine this aspect of the literature, we extracted data on what outcomes, relevant to facilitating uptake, were measured and reported, including individual level facilitators and barriers of use.

Governing Mechanisms (G) refers to findings that provide information on governance arrangements and policy settings needed to facilitate the establishment of e-mental health services within the health care system. We provided details about the policy implications of papers, classified according to a typology of policy mechanisms relevant to health governance: Organization, Regulation, Community Education, Finance, and Payment [20]. We added Information Communication Technology as a category, as this is a rapidly evolving area of health policy that may or may not be adequately encompassed by existing typologies for classifying policy mechanisms.

## Results

### Study Selection

**Figure 2:**
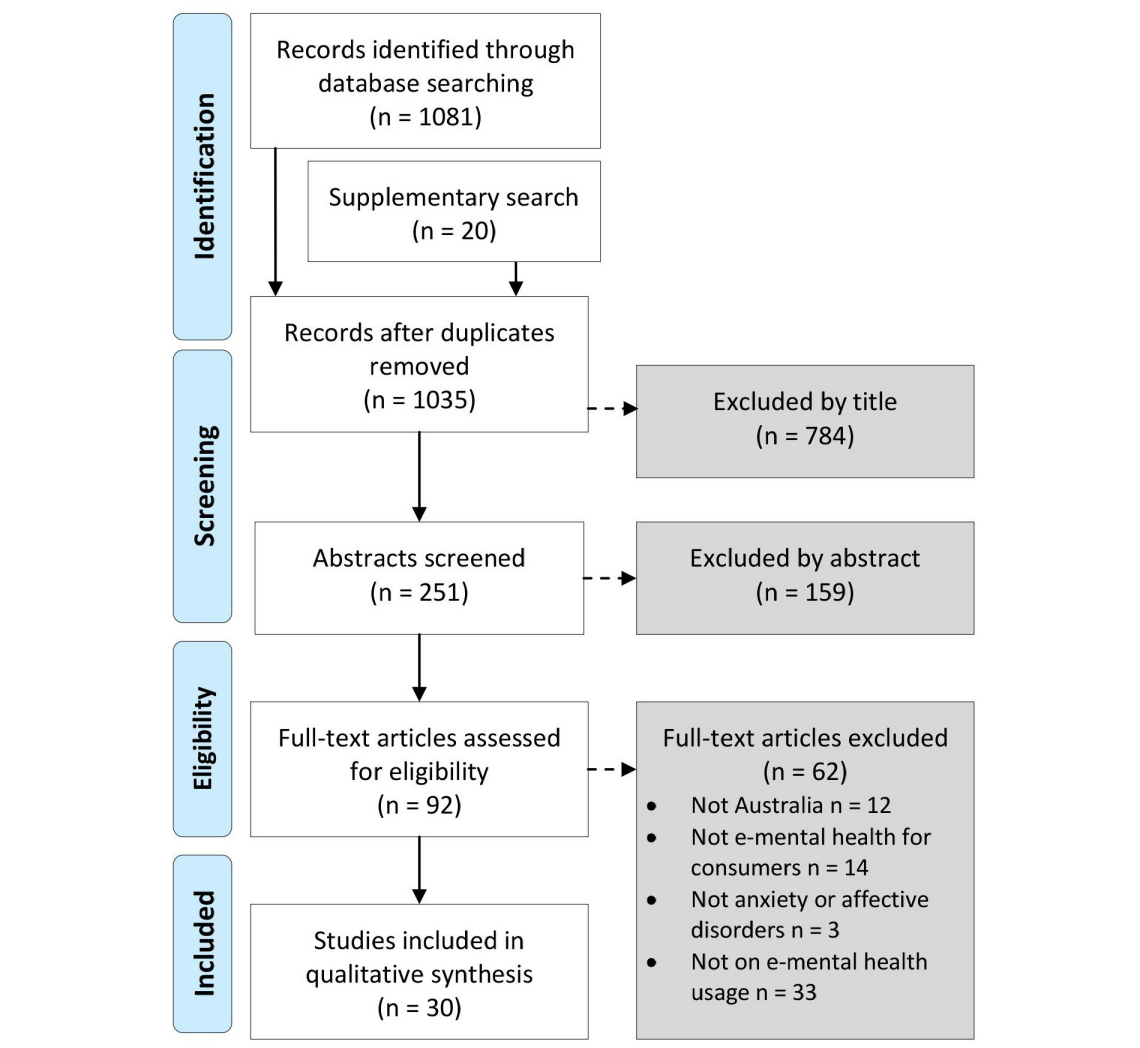
PRISMA flow diagram for study inclusion.

As shown in Figure 2, the database search yielded 1081 records, comprising 17% from Cochrane, 38% from PubMed, 7% from PsycINFO, 4% from CINAHL, 25% from Embase, and 9% from ProQuest Social Science. The supplementary search yielded an additional 20 records for consideration of which four were included in full-text screening. After duplicates were removed, 1035 records went through the title screening stage to exclude studies that were not on e-mental health (eg, studies on stroke, dementia, chronic pain, or weight management), from which 784 records were excluded, leaving 251 records for abstract screening. From screening the abstracts, 159 records were excluded, which left 92 records for full-text assessment for eligibility. A further 62 records were excluded due to the following reasons: not Australia-focused (12/62, 19%), not e-mental health for consumers (14/62, 23%), not for anxiety or depressive disorders (3/62, 5%), and not on e-mental health usage (33/62, 53%). A list of the excluded studies along with the reasons for exclusion is presented in Multimedia Appendix 1. A total of 30 studies were included in the analysis.

### Study Characteristics

Our findings show that the majority of research to date has been conducted on clinical and self-selected online help-seeking populations (see Table 1 [21-50]). From the included studies, 63% (19/30) of studies were conducted on existing or recruited e-mental health users and participants (EU), 30% (9/30) were conducted on general populations or prospective users (PU), and 10% (3/30) of studies were conducted on service providers (SP). Most empirical research (24/30, 80%) has been undertaken by the developers of the interventions being discussed. Our key focus was to draw together literature on e-mental health service use, including facilitators and barriers; however, only 60% (18/30) of the studies included e-mental health utilization as a research question. Of the included studies, 93% (28/30) were trials and online surveys (level II evidence) and the other two were qualitative interview studies (level III evidence). Sample sizes varied markedly across studies, with sample sizes ranging from 10 to 110,825. Fewer than half (13/30, 43%) of the studies were given a policy relevance rating of Low; 47% (14/30) had a rating of Mod. A small minority of studies (3/30, 10%) provided empirical evidence focused on implementation, that is, had a policy relevance rating of High. In terms of area of policy relevance, we classified 97% (29/30) of studies as relevant to understanding e-mental health Target Demographic (T), 57% (17/30) as relevant to Facilitating Uptake (F), and 77% (23/30) as relevant to Governing Mechanisms (G) of e-mental health within the health system.

**Table 1:** Study characteristics (N=30).

Reference	Study aims (Aims type, Level of evidence – Sample type)^a^	Sample size	Policy relevance: level^b^; area^c^
Anderson et al, 2012 [21]	Examine the quality of the working alliance in online cognitive behavioral therapy (CBT) for anxiety disorders in youth and the role of working alliance and compliance in predicting treatment outcome (B, II-EU)	132 children and adolescents, and their parents	Low; T
Bennett et al, 2010 [22]	Describe ehub and populations for whom it may be suited (B, II-EU)	110,825 website visitors to e-mental health site	Mod; TG
Bradford, Rickwood, 2014 [23]	Determine whether adolescents prefer online over more traditional types of mental health service delivery, what their help-seeking intentions are for a commonly experienced mood disorder and the factors that affect these intentions (A, II-PU)	231 school students	Mod; TFG
Casey et al, 2013 [24]	Assess the impact of providing e-mental health information on attitudes toward e-mental health services (A, II-PU)	217 general convenience sample	High; FG
Christensen et al, 2006 [25]	Examine predictors of depression and anxiety scores on the MoodGYM website as a function of user characteristics, and to compare the compliance rates of the original site with the new public version of the site (B, II-EU)	58,398 public registrants to the MoodGYM site	Low; T
Crisp, Griffiths, 2014 [26]	Examine the characteristics associated with interests and preferences in using online mental health interventions (A, II-PU)	4761 Well-Being Project participants	Mod; TFG
Dear et al, 2013 [27]	Evaluate the efficacy, acceptability, and feasibility of a brief iCBT^d^ program, Managing Your Mood Program, to treat depression among older adults aged 60 years and older (A, II-EU)	20 older adults	Low; TFG
Dear et al, 2015 [28]	Examine acceptability, efficacy, and health economic impact of two self-guided iCBT programs for adults over 60 years of age with anxiety and depression (A, II-EU)	47 older adults	Mod; TFG
Dingwall et al, 2015 [29]	Examine the acceptability, feasibility, and appropriateness of e-mental health resource app for use by service providers with Aboriginal and Torres Strait Islander communities (A, III-SP)	15 Aboriginal and Torres Strait Islander service providers	Mod; TFG
Donker et al, 2013 [30]	Predict treatment outcomes of new e-couch Internet-delivered Interpersonal Psychotherapy (iIPT) and CBT against MoodGYM CBT (B, II-EU)	1843 spontaneous website visitors	Low; T
Ellis et al, 2012 [31]	Explore young people’s attitudes and behaviors in relation to mental health and technology use (A, II-PU)	1038 young people	Mod; TFG
Ellis et al, 2013 [32]	Explore young men’s knowledge, attitudes, and behavior towards mental health and technology use (A, II-PU)	486 young men from online surveys and 118 from focus groups	Mod; TFG
Gun, Titov, Andrews, 2011 [33]	Explore levels of acceptability of Internet-based treatment programs for anxiety and depression (A, II-EU & SP)	1543 health professionals and lay people	High; TFG
Johnston et al, 2014 [34]	Explore the efficacy and acceptability of iCBT for young adults with anxiety and depression (A, II-EU)	18 young adults	Low; TFG
Keane et al, 2013 [35]	Examine the use of the Internet to access mental health information by demographic characteristics (A, II-PU)	2996 general population	High; TFG
Kirkpatrick et al, 2013 [36]	Report acceptability, feasibility, and preliminary efficacy of established iCBT course (Well-being Course) being administered by nongovernmental organization for anxiety (A, II-EU)	10 adult callers or website visitors of Mental Health Australia	Mod; TFG
Kiropoulos et al, 2008 [37]	Compare the effectiveness of iCBT versus face-to-face CBT for panic disorder and agoraphobia (B, II-EU)	86 people with panic disorder	Low; T
Klein et al, 2011 [38]	Evaluate the Anxiety Online programs (B, II-EU)	225 people self-selected for e-therapy programs	Low; T
Klein et al, 2010 [39]	Open trial to evaluate posttraumatic stress disorder online (B, II-EU)	22 adults with posttraumatic stress disorder	Low; T
Klein, Richards, Austin, 2006 [40]	Compare the efficacy of Internet-based self-help and self-help manual for treating panic disorders (B, II-EU)	55 people with panic disorder	Low; T
Morgan, Jorm, Mackinnon, 2012 [41]	Test the effectiveness of an automated email-based campaign promoting self-help behaviors (B, II-EU)	1326 adults with depression	Low; TG
Neil et al, 2009 [42]	Investigate adherence rates to a CBT website in adolescent samples from a school-based or community setting (A, II-EU)	1000 school-based and 7207 community-based adolescents	Mod; TFG
O'Kearney et al, 2009 [43]	Evaluate the benefits of MoodGYM compared to a usual high school curriculum (B, II-EU)	157 girls	Low; TG
Pier et al, 2008 [44]	Evaluate the efficacy of an iCBT intervention (Panic Online) for the treatment of panic disorder (B, II-EU)	65 people with panic disorder	Low; TG
Proudfoot et al, 2010 [45]	Explore community attitudes toward the appropriation of mobile phones for mental health monitoring and management (A, II-PU)	525 from online survey; 47 from focus groups; 20 interviews	Mod; TFG
Robertson et al, 2006 [46]	Test the feasibility of implementing an e-mental health system for the treatment for depression (A, II-EU)	144 depressed adults	Low; TG
Sinclair et al, 2013 [47]	Understand rural clinicians’ attitudes towards the acceptability of online mental health resources as a treatment option in the rural context (A, III-SP)	21 rural clinicians	Mod; TFG
Titov et al, 2010 [48]	Examine characteristics of adults with anxiety and depression treated at an Internet clinic with national survey data and outpatient clinic data (B, II-PU)	774 volunteers to an Internet Clinic, 454 patients in an anxiety disorders outpatient clinic, 627 National survey cases	Mod; TG
Wootton et al, 2011 [49]	Establish the acceptability of iCBT treatments for adults with obsessive compulsive disorder (A, II-PU)	129 volunteers to an online survey, 135 in an anxiety disorders outpatient clinic, 297 National survey cases	Mod; TFG
Zou et al, 2012 [50]	Perform feasibility study for iCBT for anxiety in older adults (A, II-EU)	22 older adults with anxiety	Mod; TFG

^a^Study aims type: A=includes investigation of barriers and facilitators of e-mental health use as part of the research aim; B=provides information about e-mental health use, including barriers and facilitators, even though this was not part of the research aim. Study aims level of evidence: II=randomized controlled trials, observational studies, or case-control studies; III=case series, focus groups; EU=study of existing e-mental health service users or self-selected sample; PU=study was on prospective e-mental health users; SP=study of service providers.

^b^Policy relevance level: Low=minimal policy relevance, Mod=some policy relevance, High=direct policy relevance/policy-focused.

^c^Policy relevance area: T=Target Demographic, F=Facilitating Uptake, G=Governing Mechanisms

^d^iCBT=Internet-based cognitive behavioral therapy.

### Target Demographic

The 29 studies that provided information on target demographics provided variable detail on their study samples and the characteristics of e-mental health users (see Multimedia Appendix 2). As mentioned already, there was a bias towards online recruitment and self-selected e-mental health service users. Study samples tended to be biased towards females. Program development has targeted different age groups with tailored programs, and young people have received particular attention to date (6/29, 21%). Half of the studies (15/29, 52%) provided information about socioeconomic status (mostly employment status) of e-mental health care users. Where relevant information was provided, it appeared there was a bias towards middle- to high-income earners. Nearly half of studies (13/29, 45%) provided information about educational attainment and in these there was a bias towards more highly educated members of the public. Only 14% of studies (4/29) asked or provided information about ethnicity, directly or indirectly. Inclusion/exclusion criteria for studies requiring fluency in written English, as well as technological requirements (eg, access to a computer, Internet, and printer), reinforce these biases. Only 14% of studies (4/29) cited statistics on the geographic location of participants (eg, urban versus rural), and few studies provided information on relationship status of participants.

### Facilitating Uptake

Synthesized results of the 17 studies that provided information on measurements related to facilitating uptake of e-mental health are presented in Table 2 [23,24,26-29,31-36,42,45,47,49,50] and Multimedia Appendix 3. As detailed earlier, the majority of research included in this review was designed to justify, or enhance, the efficacy and effectiveness of online interventions rather than to investigate the appeal of currently available online therapies as a possible course of treatment for prospective help-seekers (see Multimedia Appendix 3). Consequently, many of these studies were focused on enhancing uptake through program development rather than investigating how systems-wide implementation could be achieved via policy and planning. Having said this, satisfaction with treatment was linked to likelihood of recommending e-mental health to others in a number of studies [27,28,34,36,50].

**Table 2:** Facilitators and barriers for e-mental health utilization (N=17).

Reference	E-therapy utilization: Facilitators	E-therapy utilization: Barriers	Non-significant factors
[23]	Motivated to seek face-to-face help rather than receive no help	Not preferring online treatment	Self-reliance
		Females prefer face-to-face help	Shyness
	Males who would have otherwise chosen no help	Lower mental health literacy	Stigma
	Higher mental health literacy	Viewing e-therapy as impersonal	
	Anonymity of the Internet	Lack of trust	
	Accessibility of information	Not knowing who you are talking to	
	Connecting with others who have been through the same thing	Lack of customized feedback	
[24]	Knowledge about e-mental health through provision of textual information	Lack of knowledge about e-mental health	Type of e-mental health service
		Attitude that online programs without therapist assistance are not helpful	
[26]	Female	Male	—
	Higher education	Low education	
	Not married	Young age	
	History of depression	Lack of interest	
	Higher depressive symptoms	Stigma	
	More free time	Too busy	
		Prefer to deal alone	
[27]	High adherence	—	—
	High satisfaction linked to likelihood of recommending to others		
[28]	High satisfaction linked to likelihood of recommending to others	—	—
[29]	Attractive visual appeal	Technology issues	Individual mental health issues
	Ease of use	Time constraints for service providers	Age
	Culturally appropriate	Concern for job security	Sex
	Enjoyable / fun	Translation into Indigenous languages	
	Appropriate training for service providers		
[31]	Positive attitudes towards e-mental health in general	Male	—
		Interactive games were not preferred	
[32]	Privacy and anonymity	Ideas about masculinity	—
		Preference for reliance on informal networks	
		Preference for self-help	
		Generalized scepticism of “interventions”	
[33]	Low severity of mental health symptoms	Lack of information about effectiveness of e-mental health	The need for reliable Internet
		Lack of knowledge about treatments available	Lack of computer skills
		Lack of established guidelines	IT support
		Unclear about legal issues involved or liabilities of recommending e-therapies	
		Lack of training for health professionals	
		Preference for not seeking help at all over using e-mental health	
		Lack of experience in using e-mental health treatments	
[34]	Good adherence	—	Low acceptability
	High satisfaction, linked to likelihood of recommending e-mental health		
[35]	Female	Male	Metropolitan versus rural location of residence
	Younger age (15-54)	Older age	
		Low overall usage	
[36]	High satisfaction	Therapist initial scepticism	—
[42]	Monitored settings, such as school-based settings	Unmonitored-settings	History of depression
	Female	Male	
	Living in rural areas		
[45]	Symptoms of depression, anxiety, or stress were more likely to be interested in mobile mental health	Perceived as not helpful	Sex
		Negative attitudes towards technology	Age
	Speed and convenience	Privacy concerns	Employment
	Ease of access	Lack of Internet access on mobile phone	Marital status
	Positive attitude towards self-help	Small screen of mobile phone	
	At least some access		
	Less confronting than face-to-face-consultation		
[47]	Usability, privacy	Inadequate (private) Internet access in some rural settings	—
	Provides some services to rural areas where there is a lack of service	Reading difficulties among consumers	
	Training for clinicians	Computer literacy	
	Provision of informational materials for providers and consumers	Difficulty accessing training in the rural environment	
	Ability for e-mental health to be integrated with existing care	Practitioner concerns about lack of feedback from clients, rumination or social isolation	
	Promotion of e-mental health as an effective treatment	Scepticism about the effectiveness of e-mental health treatments	
		Lack of time to explore resources	
[49]	Embarrassment of face-to-face	Prefer face-to-face	Got told not to use
	Believed that e-mental health would be useful	Embarrassment	Lack of access to computer/Internet
	Privacy and anonymity	Perceived as not effective	
	Convenience	Cannot see a person	
	Bridges travel issues	Inferior to communication with therapist	
	Reduced costs	Do not know what e-mental health care is	
	Willingness to try	Prefer self-management	
	Useful for mild symptoms	Too confronting	
		Problems not severe enough	
		Prefer medications	
		Sounds too risky	
		Lack of time	

[50]	High level of satisfaction, related to likelihood of recommending treatment to a friend.	—	—

Six of 30 studies (20%) sought to understand treatment preferences for online therapies compared to face-to-face psychological therapies, including “interest” or “willingness to try” online therapies [23,31-33,45,49]. We did not find studies that directly compared preferences for online therapies, face-to-face therapies, and pharmacotherapies. Two studies quantified relative preferences [23,33] and found a preference for face-to-face therapies over online therapies. In a sample of adults who visited a website for depressive and anxiety disorders, 63% of participants preferred face-to-face, compared to 7% who preferred e-mental health services [33]. Similarly, in a non-clinical sample of students in grades 10-12 recruited from schools, 58% preferred face-to-face, compared to 16% who preferred e-mental health services [23]. There was some indication that online therapies with practitioner support were preferred to online-only therapies [23,47]. The exception to this rule was that young men preferred informational websites to treatment-oriented websites [31].

Facilitators and barriers for e-mental health utilization are presented in Table 2. Stigma, broadly defined, was highlighted as both facilitating the use of e-therapies (including, “embarrassment” of seeking face-to-face help), as a barrier to use, and as non-significant [23,26,49]. Mental health literacy was highlighted as a facilitator in one study [23], and awareness (or lack thereof) of e-mental health was identified as important in four studies [24,32,47,49]. Being a rural resident was identified as a facilitator [42], a barrier [47] and as non-significant [35]. Some perceived qualities of e-mental health care were both facilitators and barriers, depending on whether different individuals interpreted them positively or negatively. For example, some studies identified “anonymity” as a facilitator of e-mental health use [23,47,49], but anonymity was arguably also a barrier when e-mental health services were seen as depersonalized [23,45]. Assessments about using e-mental health care differed depending on different beliefs as to whether important requirements, such as the need for privacy, were met. For example, concerns with privacy could be a facilitator of use, if e-mental health care was perceived as private [47,49]. However, “concern with privacy” was also deemed a barrier to use [45], indicating that some people do not perceive e-mental health care as protecting privacy. A preference for “self-help” was also reported as being a barrier or facilitator to the use of e-mental health [23,26,32,49], depending on whether e-mental health was viewed as consistent with self-help or not. Both lower symptom severity [33] and higher symptom severity [26,45] have been identified as facilitators of use.

### Governing Mechanisms

Over three-quarters (23/30, 77%) of studies examined factors from which we could draw inferences about policy mechanisms needed to establish e-mental health within the health system (see Table 3 [22-24,26-29,31-36,41-50]). However, none of these studies characterized the policy settings required to implement e-therapies. Nineteen of these studies (83%) provided insight into the organizational requirements for establishing e-mental health. These described settings in which e-mental health could justifiably be implemented, namely, schools, general practice, non-governmental mental health organization websites, and through direct-to-public online delivery. These studies also described configurations of e-mental health care delivery (eg, informational websites, peer support websites, Internet-only therapy or clinician-moderated e-mental health care) that may be best accepted by different sectors of the population. However, these studies did not provide details on the relative merits of implementation of different organizational types at scale, nor how implementation in different settings might occur.

**Table 3:** Governing mechanisms (N=23).

Reference	Implications for governing mechanisms	Details related to governing mechanisms^a^
[22]	Organization	Justifies the provision of Internet-only therapy.
[23]	Organization, Community education	Quantifies preferences among young people for online help, face-to-face help, and tele-help.
		Identifies factors that may influence appeal of online help via health promotion.
[24]	Community education	Identifies text-based methods as best means of delivering information about e-mental health.
[26]	Finance/payment	The paper itself does not make the following argument; however, the paper identifies that financial incentives could nudge approximately 20% of participants to engage with e-mental health.
[27]	Organization	Establishes feasibility and acceptability of iCBT for adults 60 years and over with depression.
[28]	Organization, Finance/payment	Establishes feasibility and acceptability of iCBT for adults over 60 years old with depression and anxiety.
		Quantifies economic health costs associated with participating in the programs at around $60 per person.
[29]	Organization, Community education, Information communication technology	Highlights the feasibility and acceptability of service providers in remote Aboriginal and Torres Strait Islander communities using mobile apps to engage with consumers.
		Highlights the need for training and informational materials for service providers.
		Highlights infrastructural and technical barriers to information communication technology use in remote areas.
[31]	Organization, Community education	Showed that young people preferred websites with information or online clinics to websites with question and answer or interactive games.
[32]	Organization, Community education	Suggests tailoring online services (informational and treatment) to different tastes.
[33]	Regulation, Organization, Community education, Information communication technology	Quantifies preferences for Internet treatment compared with face-to-face treatments.
		Identifies concerns with liability as an issue for health professionals recommending Internet-based treatments.
		Identifies health professionals’ and lay persons’ needs for more information about Internet-based treatments, including information about effectiveness.
		Identifies infrastructure and computer literacy as barriers to use among a minority of health professionals and lay people.
[34]	Organization	Justifies feasibility of Internet-only therapy for young people.
[35]	Community education	Highlights (and quantifies) characteristics of potential user groups for e-mental health. Middle-aged rural females most disposed, older rural males least disposed.
[36]	Organization, Community education	Justifies feasibility of delivering iCBT via not-for-profit organizations’ websites.
		Registered clinicians not necessary for delivery, can train other staff.
[41]	Community education	Internet-delivered self-help messages are a low-cost, automated, and easily disseminated prevention option.
[42]	Organization	Justifies school-based delivery of online interventions for depressive and anxiety disorders for adolescents.
[43]	Organization	Justifies delivery of MoodGYM in school settings.
[44]	Organization	Justifies delivery of iCBT for panic disorder with either face-to-face support from general practitioner or email support from psychologist.
[45]	Organization, Regulation, Information communication technology	Privacy and security are important to people using mobile health.
		Not suitable for those who dislike the use of technology.
		Highlights feasibility of mobile mental health.
[46]	Organization	Justifies use of comprehensive eHealth system for management of depression, including adherence to medication (including consultations, monitoring, psychoeducation, and therapy).
[47]	Organization, Community education	Overall, rural clinicians supported implementation of Internet-assisted therapies, as an adjunct to face-to-face consultations.
		Highlights need for informational materials for rural clinicians and consumers.
[48]	Organization	Justifies iCBT for anxiety and depressive disorders for the wider population.
[49]	Organization, Regulation	Justifies demand for Internet-based treatments for obsessive compulsive disorder.
		Privacy and anonymity important to using face-to-face treatment.
[50]	Organization	Justifies feasibility of iCBT for older adults with anxiety.

^a^iCBT=Internet-based cognitive behavioral therapy.

Ten studies (10/23, 43%) provided insights on community education. One study investigated the usefulness of different modes of delivery of information (eg, by text or by film) about e-mental health care and found that providing text-based information increased likelihood to use e-mental health services in the future [24]. Studies that included information about service providers’ views highlighted the need for informational materials and training about e-mental health, including evidence about its efficacy and also the need to distribute information about liability.

Two studies provided some information relevant to financing and payment [26,28]. One study provided an estimate of total health care costs associated with using Internet-based cognitive behavioral therapy (iCBT), showing that iCBT use was associated with marginally higher health care costs [28]. The other identified that participants’ willingness to complete iCBT interventions might be enhanced by appropriate financial incentives (ie, nudges) [26].

Two studies addressed regulatory issues. These included participants’ concerns about privacy and anonymity [45], which has relevance to data collection, storage, and security, and health care professionals’ concerns about legal liability [33] for recommending and using Internet-based treatments. Finally, three studies highlighted infrastructure and technical issues [29,33,45] associated with deploying mobile-health technologies, including in remote or Aboriginal and Torres Strait Islander communities [29,33]. Computer literacy was seen as a minor issue [33].

## Discussion

### Principal Findings

Meta-analyses show that Internet-based and Internet-assisted therapies are an effective means of treating many individuals with depressive and anxiety disorders, and that those who use these therapies tend to be satisfied with them [1]. While these results show that e-mental health has a potentially important role in the Australian health system, the evidence base does not adequately define the population for whom e-mental health care is, and could be, most suitable. It does not accurately benchmark current use or provide indications of likely future levels of e-service use compared to other treatments. It also does not present sufficient information to inform policies that could facilitate its broad-scale adoption. These findings corroborate a recent review and NHMRC Case for Action [7,9], a review that found no policy-focused research has been undertaken on e-mental health [19] and calls for further translational research in this area [51].

Current knowledge on determinants of e-mental health service use presents a program development perspective on e-mental health establishment. The primary focus of proposed translational activities has been on closing the evidence-practice gap, ensuring the viability of e-services through financing, and enhancing the reach of, and adherence to, e-services including through promotion [9]. These are important and necessary translational activities. However, facilitating the establishment of e-mental health care within the Australian health system requires additional translational research to provide, what we term, a “policy-making” perspective. Distinctively from translational research activities focused on consolidating and expanding e-services within the Australian health system [9], a policy-making perspective approaches the question of implementing e-mental health, exogenously, based on two primary considerations: (1) the kinds of mechanisms available to government to facilitate implementation and (2) the imperative to fit e-mental health care within a population-based, stepped-care model that includes a range of treatment types for depressive and anxiety disorders and incorporates contingency planning.

The studies we reviewed were mostly clinical trials conducted with self-selected e-therapy users. Information about culture/ethnicity and socioeconomic status are infrequently reported. Based on the studies we reviewed, there seems to be a sex bias, with females more likely to use e-mental health care than males. These patterns of use probably reflect patterns of utilization in face-to-face treatment seeking [52]. Highlighting these biases does not undermine the value of e-services but is important to ensuring that integrating e-services into the mental health system works to overcome inequalities, rather than exacerbate them. How best to respond to these biases is unclear, as three courses of action are possible: (1) invest in promoting existing e-services to under-using demographics, (2) design new services tailored for these populations, and (3) invest in funding alternative treatment modalities that may be more attractive to groups who underutilize e-services. Further policy-focused research on non-use of e-mental health care is important to informing appropriate future courses of action with respect to these biases.

Different studies investigated and reported different possible facilitators and barriers to use and the concepts investigated proved to be fairly slippery. Factors that may facilitate or impede use operate at different scales and levels and can be viewed differently from different perspectives. In other words, constructs can be worded as both facilitators and barriers while reflecting a similar process. Additionally, there was evident symmetry as to what is a facilitator or barrier. For example, different beliefs about whether or not online therapies are private as well as whether or not anonymity is an appealing or undesirable quality in a treatment, highlights the importance of different interpretations as well as preferences. While lack of consistency in the definition of constructs across studies likely contributes to a lack of unequivocally identifiable facilitators or barriers, we think that further diverse examination of facilitators and barriers is needed before any calls for standardization of constructs is warranted. More pressing is the need to examine different interpretations of online health interventions to inform the detail (wording) of community education campaigns.

### Future Research Directions

Policy-focused research is required to (1) prioritize ongoing research and development of e-services that will ensure adequate coverage of mental health care for prospective e-help-seekers, (2) provide accurate estimates of current e-mental health usage and identify realistic future targets for e-service use, relative to other service and treatment types, (3) elucidate the factors underlying preferences for and against therapies, particularly to inform promotional materials that resonate with different perceptions and values of self-help, privacy, and anonymity, and (4) inform the establishment of appropriate governing mechanisms for e-services, giving highest priority to privacy and data security, liability, and modes of financing and payment [9].

Conducting this research independently of e-mental health program development will allow for resourcing across research and development and service delivery to be informed by a critical appraisal that includes contingency planning. Research focused on increasing adoption and adherence is focused on engaging with consumer preferences as well [9]. However, from a policy-making perspective, understanding preferences, and how malleable these might be, has a slightly different function insofar as it can inform decisions about how to allocate funds to different activities along the translational spectrum from program development to promotion. Understanding preferences is also important in deciding how to allocate resources to other treatment modalities and institutions that address the downstream impacts for those who, for whatever reason, remain untreated.

Methodologically speaking, in addition to translational research identified elsewhere [9], we recommend:

1. Further reviews of eHealth policy from Australia and internationally to inform policies on privacy, data security, liability, and modes of financing and payment for services. These reviews should draw on academic and gray literature across a range of eHealth and telehealth areas, with the aim of identifying suitable regulatory mechanisms for governing e-mental health. Literature reviews can be enhanced through stakeholder interviews with Australian e-service developers and providers as well as policy makers.
2. Qualitative interview studies of current users and non-users of e-mental health services, including semi-structured interviews and think-aloud exercises, should be conducted to inform the details of promotional materials that will resonate with disparate perceptions of e-mental health services with respect to issues of stigma, privacy, anonymity, and self-help.
3. Surveys using discrete choice experiment methodologies are important for accurately characterizing preferences for e-mental health care, face-to-face therapies, and prescription medications. Prescription medications, in particular, are the “elephant in the room” of e-mental health studies; including this treatment in comparisons is important given the biases evident in e-mental health care use and in understanding the scope and limits of e-mental health care for those who are not fluent in English or have low literacy or comprehension. A course of prescription medications has minimum language or comprehension requirements.

### Limitations

We elected to focus on Australian research because policy development is importantly context-specific [14]. Nonetheless, our conceptual framework and methodological approach for this study and the implications drawn for future research all have international relevance. The inferences made under the theme “Target Demographic” must be understood in relation to our search criteria, which focused on factors influencing service use and thus did not include feasibility or effectiveness studies for programs targeting different cultural groups that did not provide data on service use factors [53-55]. Policy studies and economics research relevant to the topic may have been excluded because they are not found in medical databases searched. Our capacity to undertake truly multidisciplinary systematic reviews may have been limited by differences in the meanings of words in medical research versus political and social sciences, the specificities of MeSH terms, and other conventions for identifying search terms, and the different framings and focus of research in different disciplines. However, we attempted to overcome this limitation by searching databases such as ProQuest Social Science and search engines like Google Scholar. In addition, our search was conducted using a combination of headings as well as keywords and synonyms across the different disciplines. Our findings corroborate other reviews that point to a lack of translational research in this area. Therefore, we are reasonably confident about our results.

Our classification scheme for policy-relevant research does not acknowledge the “behind-the-scenes” development of implementation-focused thinking that can inform research design and questions nor policy advocacy work that addresses implementation issues. Finally, our review excluded general e-mental health studies (eg, [56]) that focused on service use types but did not investigate disorder type specifically, as the scope of our review included only depressive and anxiety disorders. We do not think such omissions invalidate our conclusions.

### Conclusion

Successfully establishing e-mental health care within the health system will depend on the skillful coordination of activities within clinical, community, research and development, and policy-making realms. This, in turn, will depend on appropriate translational research being conducted that is relevant to each of these domains. This review provides a rationale and framework for undertaking dedicated policy-focused research on e-mental health in the future.

## Multimedia Appendix 1

List of excluded studies with exclusion reasons.

## Multimedia Appendix 2

Target demographic.

## Multimedia Appendix 3

Measurements related to facilitating uptake and evidence on e-mental health utilization.

## Abbreviations

CBT: cognitive behavioral therapy
EU: study of existing e-mental health service users or self-selected sample
F: facilitating uptake
G: governing mechanisms
iCBT: Internet-based cognitive behavioral therapy
iIPT: Internet-based interpersonal therapy
Mod: Moderate
NHMRC: National Health and Medical Research Council
PU: study was on prospective e-mental health users
SP: study of service providers
T: target demographic
