# Supplementary material for: Establishing and Governing e-Mental Health Care in Australia: A Systematic Review of Challenges and A Call For Policy-Focussed Research
Source: J Med Internet Res. 2016 Jan 13;18(1):e10. doi: 10.2196/jmir.4827 (PMC4730106; doi:10.2196/jmir.4827)
Supplement: Supplementary file 1 [file jmir_v18i1e10_app1.pdf]

Multimedia Appendix 1. List of excluded studies with exclusion reasons (n = 62).

Excluded studies
**Not Australia focused (n = 12, 19%)**
Andrews G, Cuijpers P, Craske MG et al., 2010; Carter FA, Bell CJ, Colhoun HC, 2013; Clarke AM, Kuosmanen T, Barry MM, 2015; Dyck KG, Hardy C, 2013; Gibson KL, Coulson H, Miles R et al., 2011; Hoifodt RS, Lillevoll KR, Griffiths KM et al., 2013; Johnson GR, 2014; Knowles SE, Toms G, Sanders C et al., 2014; Melville KM, Casey LM, Kavanagh DJ, 2010; Prentice JL, Dobson KS, 2014; Volpe T, Boydell KM, Pignatiello A, 2014; Wuthrich VM, Rapee RM, Cunningham MJ et al., 2012
**Not about e-mental health treatments or interventions for consumers (n = 14, 23%)**
Costin DL, Mackinnon AJ, Griffiths KM et al., 2009; Cunningham M, Rapee R, Lyneham H, 2006; Donovan CL, March S, 2014; Hickie IB, Luscombe GM, Davenport TA et al., 2007; Kauer SD, Reid SC, Crooke AH et al., 2012; Kingston D, McDonald S, Tough S et al., 2014; Martin A, Sanderson K, Scott J et al., 2009; Mattison M, 2012; Morgan AJ, Jorm AF, Mackinnon AJ, 2013; Parker G, Fletcher K, Barrett M et al., 2006; Ramos-Ríos R, Mateos R, Lojo D et al., 2012; Sansom-Daly UM, Wakefield CE, Bryant RA et al., 2012
**Not designed to treat depressive or anxiety disorders (n = 3, 5%)**
Kay-Lambkin FJ, Baker AL, Kelly B et al., 2011; Klein B, Cook S, 2010; Milgrom J, Gemmill A, 2014
**No empirical evidence provided on e-mental health usage (n = 33, 53%)**
Andrews G, Titov N, 2010; Burns J, Birrell E, 2014; Christensen H, Hickie IB, 2010 (A); Christensen H, Hickie IB, 2010 (B); Christensen H, Petrie K, 2013; Christensen H, Reynolds J, Griffiths KM, 2011; Clarke J, Proudfoot J, Birch MR et al., 2014; Cleary M, Walter G, Matheson S, 2008; Clough BA, Casey LM, 2011; Dear BF, Titov N, Schwencke G et al., 2011; Dear BF, Zou J, Titov N et al., 2013; Griffiths KM, Christensen H, 2007; Harrison V, Proudfoot J, Wee PP et al., 2011; Johnston L, Titov N, Andrews G et al., 2013; Lal S, Adair CE, 2014; Lauder S, Chester A, Castle D et al., 2013; Leeder S, 2015; Lind C, Boschen MJ, Morrissey S, 2013; Mahoney AEJ, Mackenzie A, Williams AD et al., 2014; Mewton L, Hobbs MJ, Sunderland M et al., 2014; O'Kearney R, Kang K, Gibson M et al., 2007; Proudfoot J, 2013; Robinson J, Hetrick S, Cox G et al., 2014; Sunderland M, Wong N, Hilvert-Bruce Z et al., 2012; Titov N, 2007; Titov N, Dear BF, Johnston L et al., 2013; Titov N, Dear BF, Schwencke G et al., 2011; Wallace D, Rayner S, 2013; Watts S, Mackenzie A, Thomas C et al., 2013; Watts S, Newby JM, Mewton L et al., 2012; Williams AD, Thompson J, Andrews G, 2013; Wood J, Stathis S, Smith A et al., 2012; Wootton BM, Titov N, 2010
