# Supplementary material for: Establishing and Governing e-Mental Health Care in Australia: A Systematic Review of Challenges and A Call For Policy-Focussed Research
Source: J Med Internet Res. 2016 Jan 13;18(1):e10. doi: 10.2196/jmir.4827 (PMC4730106; doi:10.2196/jmir.4827)
Supplement: Supplementary file 2 [file jmir_v18i1e10_app2.pdf]

Multimedia Appendix 2. Target demographic (N = 29).

**Sample characteristics (categories of characteristics referred to in text)**	**Inclusion criteria | Means of recruitment**	**Mental disorder targeted (Prevention or Treatment)**	**Platform or mode of e-mental health service**
[21] Children and adolescents aged 7-18 years, and their parents; 88% Born in Australia, 79% lived with biological parents, compared to general population sample was from mid-high income families and relatively well educated (SEX, AGE – YP, EDU, SE, ETH)	Age >=10; computer and internet access at home | Referral from mental health professionals and media	Anxiety (T)	BRAVE minimally therapist-assisted online CBT program; 10 youth sessions; 5 parent sessions; booster sessions at 1 and 3 months
[22] Website-registrants to the e-mental health sites, MoodGYM, e-couch, BlueBoard, and BluePages (SEX, AGE)	n/a | All registrants	ehub self-help for mental health and wellbeing (P & T)	The ehub resources: MoodGYM, e-couch, BlueBoard, BluePages.
[23] Students from public and private schools; aged 15 to 19 years; 60% females; 57% private schools; grade 10-12 (SEX, AGE – YP)	Non-clinical sample | Students were approached and participated as volunteers	Commonly experienced mood disorders (P)	Help online, for a vignette describing depression
[25] Public registrants to the MoodGYM site (SEX)	Complete data across the program | Site visitors and MoodGYM participants	Depression and anxiety (T)	MoodGYM self-guided iCBT; 5 modules
[26] Well-Being Project participants with internet access; 63% females; 71% married; 49% with history of depression; 19% current depression (SEX, AGE)	Age >=18; psychologically distressed; home or work internet access; not receiving CBT or treatment from mental health professional; not participating support groups; no history of psychosis, schizophrenia, or bipolar; completion of key demographic questions | Random	Depression (T)	Information (HealthWatch); internet interventions (E-couch); online support group (ISG); or combination

	sample from the Australian Electoral Roll were mailed invitations to participate		
[27] Adults aged 60 years and older, who applied online through a clinical research website on iCBT, younger end of 'elderly', and sample were more highly educated, 80% had received some form of mental health treatment in the past (AGE – 60+, EDU)	Australian residence; age >=60; access to computer Internet, and printer; at least mild anxiety as a mental disorder; not participating in CBT; not using illicit drugs or consuming >3 standard drinks/day; not schizophrenia or bi-polar; not severe depression; not taking benzodiazepine | Applicants applied online through a clinical research website for iCBT trails	Depression (T)	iCBT - Managing Your Mood Program; 5 lessons
[28] Older adults with self-report difficulties with anxiety or depression; aged >60 years; Anxiety Trial: 48% female; 74% married/defacto; 55% diploma/degree education; 52% not working; 52% previous mental health treatment; 33% on mental health medication; 54% comorbid; Depression Trial: 70% female; 30% married/defacto; 40% diploma/degree education; 90% not working; 55% previous mental health treatment; 45% on mental health medication; 30% comorbid (SEX, EDU, SE)	Australian residence; age >=60; access to computer Internet, and printer; anxiety not caused by physical illness; self-reported anxiety or depression; not participating in CBT; not using illicit drugs or consuming >3 standard drinks/day; not schizophrenia or bi-polar; not severe depression; not taking medication for anxiety or depression | Interested older adults applied online to participate in the RCTs	Depression and anxiety (T)	Self-guided iCBT without clinician contact: Managing Stress and Anxiety Course (Anxiety Trial) and the Managing Your Mood Course (Depression Trial)

[29] Service providers working with Aboriginal and Torres Strait Islanders, including health professionals, managers, program coordinators, and an Aboriginal elder (ETH)	Health professionals working with Aboriginal and Torres Strait Islander clients in Northern Territory | Purposive sampling, identification through existing professional networks including the project Expert Reference Group	Aboriginal and Torres Strait Islander mental health (T)	AIMHi 'Stay Strong' App for use by service providers with Aboriginal and Torres Strait Islander to promote mental health/wellbeing; assisted application
[30] Spontaneous website visitors; most commonly 25-39 years of age; >70% female; >85% tertiary education; around 50% partnered (SEX, AGE, EDU)	Age >=18; not receiving treatment by mental health specialists; not mental health professionals, researchers, or students | Spontaneous visitors to eCouch	Depression (T)	Self-guided iCBT eCouch and iIPT eCouch, compared to MoodGYM; 4 weeks with automated email reminders
[31] Young people aged 16-24 years; 53% female; 2.2% Aboriginal and/or Torres Strait Islander; 21% spoke a language other than English at home; 64% full-time students; 16% full-time employed<br>(SEX, AGE – YP, SE, EDU, ETH)	Young people aged 16-24 | Online snow-ball sampling using an advertisement placed on Facebook, youth serving organisations, youth centres and clinics, online service providers, charities, colleges, universities and relevant government organisations	Mental health problem (P & T)	Various and different modes of e-mental health for information, help, or support
[32] General population sample, 3% Aboriginal and/or Torres Strait Islander, 22% spoke a language other than English at home, 66% full-time students, 14% employed full-time, 9% employed part-time<br>(SEX, SE, ETH)	Young men aged 16-24 years | Online snow-ball sampling using an advertisement placed on Facebook, youth serving organisations, youth centres and clinics, online service providers, charities, colleges, universities and relevant government organisations	Mental health problems, general (P & T)	E-mental health use investigated in context of broader help-seeking. Online options provided: Website with information and/or factsheets, website with a question and answer service that sends short message service (SMS) or emails, website with online clinic,

			interactive single player games teaching life skills, interactive multiplayer games teaching life skills, and not a website.
[33] Clinical Research Unit for Anxiety and Depression (CRUfAD) visitors, including health professionals and lay people; 69% female; spending 18.29 hours per week online (SD = 15.76); 5% previously used e-mental health treatments<br>(SEX)	Age >=18; Australian residents; completed full survey | Online websites and e-news	Depression and anxiety (T)	Internet treatment; may involve completing a structured set of lessons or modules online, and/or working with a therapist online
[34] Young adults aged 18-24; 78% female; 17% married/defacto; 6% unemployed; 22% university or above education; 61% had previous mental health treatment; 17% on mental health medications<br>(SEX, AGE – YP, EDU, SE)	Age 18-24; resided in Australia; access to Internet; not psychotic or severe depression; not receiving CBT; experiencing mild depression or anxiety | Volunteer applicants through a clinical research website	Depression and anxiety (T)	Mood Mechanic Course: Therapist-guided Internet-delivered treatments for anxiety and depression; four lessons
[35] General population sample; 51% female; >=15 years; 74% metropolitan residence; 68% use the internet; 37% working full-time; 18% degree education; 63% married/defacto<br>(SEX, AGE, EDU, SE, U-R)	n/a | 2008 South Australian Health Omnibus population survey	Depression, anxiety, or relationship problems (P & T)	Online information on emotional issues
[36] Adult callers of Mental Health Australia or visitors to their online site<br>(SEX)	Resident of Australia, age 18-64, not receiving CBT, mild anxiety, access to computer, internet and printer | Mental Health Australia visitors were invited to participate	Course (T)	The Wellbeing Course, iCBT provided by Mental Health Australia; 5 lessons; 8 weeks; with telephone or email clinician contact
[37] People with panic disorder diagnosis; age 18-70 years; mean education 12.53 years (SD = 6.14); >70% female; 59% married; 63% professional occupation; 59% panic disorder with agoraphobia; 56%	Age 18-70; Australian residents; living in Victoria; primary diagnosis is panic disorder; not receiving other therapy | Panic	Panic disorder and agoraphobia (T)	iCBT: Panic Online internet-based program, email therapist assisted; 4 modules

using medications (SEX, AGE, SE)	Online website through search engines, hyper-links from mental health websites, and media releases		
[38] People self-selected for the e-therapy programs; 31% men; 96% Australian residents; 40% married; 61% metropolitan residence; 67% university or higher education; 12% unemployed; 25% on antidepressants or benzodiazepine; 42% receiving mental health assistance (SEX, EDU, SE, U-R)	Age >=18; anxiety as primary diagnosis | Facebook advertisements, referral links on mental health websites, local and national media, and presentations and brochure mail-outs to health care practitioners and consumer groups	Five main anxiety disorder types: generalized anxiety disorder (GAD), panic disorder with or without agoraphobia (PD/A), obsessive–compulsive disorder (OCD), posttraumatic stress disorder (PTSD), and social anxiety disorder (SAD) (T)	Unmoderated; Anxiety Online; 12 modules over 12 weeks
[39] Adults with primary diagnosis of PTSD; 77% females; mean education of 13.3 years (SD = 3.5); 9% unemployed (SEX, EDU, SE)	Age >=18; Australian residents; primary diagnosis PTSD; stable medication for at least 3 months; not receiving psychological treatment; not severe dissociative symptoms, severe depression and/or suicide risk, or psychosis | Australian mental health websites and local and national media	PTSD (T)	Email therapist-assisted iCBT; PTSD Online; 10 weeks
[40] People with diagnosed panic disorder; 80% female; 82% with agoraphobia; 53% on psychotropic medications (SEX)	Age 18-70; Australian residence; primary diagnosis of panic disorder; no seizure disorder, stroke, schizophrenia, organic brain syndrome, heart condition, alcohol or drug dependency, or	Panic disorder (T)	Therapist-assisted: Panic Online iCBT with email contact, MAN therapist-assisted CBT manual workbook with telephone contact, Panic Resource online information with

	chronic hypertension; not receiving other therapy or self-help | Panic Online website through search engines, hyper-links from mental health websites, and media releases		telephone contact; 6 weeks; 4 modules
[41] Adults with mild depression; 78% female; 50% Australian; 48% bachelor or above education; 50% history of depression; 3% history of bipolar or psychotic disorder (SEX, EDU)	Age >=18; mild depression; not receiving treatment for depression from health professional; resident of Australia, New Zealand, UK, Ireland, Canada or the USA; access to internet at least weekly | Internet recruitment, advertising with Google, Yahoo!, Facebook, internet forums, email newsletters, links from websites	Depression (T)	Automated emails containing advice on self-help strategies; Mood Memos; twice a week over 6 weeks
[42] School-based sample: aged 13-17; 60% female; 19% rural; 29% history of depression; Community sample: aged <=19; 72% female; 19% rural; 66% history of depression (SEX, AGE – YP, U-R)	Age <=19 | School sample: schools in the YouthMood Project; Community sample: registered on the MoodGYM public site	Depression and anxiety (P & T)	MoodGYM self-guided iCBT; 5 weeks
[43] Girls aged 15-16 years; moderate to high-income families (SEX, AGE – YP, SE)	Year 10 girls | Girls only school in Canberra	Depression and anxiety (P & T)	MoodGYM self-guided iCBT; 6 weeks
[44] People with a primary diagnosis of panic disorder; 74% female (SEX)	Fluent in English; exclusion for certain physical illnesses; not undergoing other psychological/self-help therapies; excluded if unstable medication use | Recruited from general population through local and national media, general	Panic disorder (T)	iCBT with face-to-face GP support or email psychologist support; 12 weeks

	practice, and online through mental health websites		
[45] Age >=18; Online Survey sample: 68% female; 4% unemployed; 53% married/defacto; Focus Group sample: 70% female; 6% unemployed; 29% married/defacto; Interview sample: 60% female; 10% unemployed; 45% married/defacto<br>(SEX, SE)	Unique visitors; age >=18; live in Australia | Advertised through Facebook, University of NSW website, Black Dog Institute website; intranets of various companies and consumer organisations	Mood or anxiety (T)	Mobile phone monitoring and self-help
[46] Depressed patients; age 19-64; 82% private patients; 63% female; (SEX)	Age >=18; receiving treatment for depression; depression; access to computer and internet; sufficient cognitive functioning and English | Clinicians referral	Depression (T)	RecoveryRoad; e-consultations, monitoring, psychoeducation, and therapy; 12 sessions over 12 months
[47] Rural clinicians, including general practitioners, psychologists, psychiatrists, and clinical social workers<br>(U-R)	Rural mental health specialists | Invitations sent to community-based rural mental health organizations and convenience sampling of rural mental health specialists	Mental health problems, general (P & T)	Online mental health resources general
[48] Internet clinic sample were aged 18 years or over, 34% males, 54% married/defacto, 59% with diploma/degree or above education, 68% employed, 61% ever used internet for mental health help or information<br>(SEX, EDU, SE)	Age >=18, with depression, GAD, panic disorder, or social phobia | Internet clinic sample recruited from iCBT programs at VirtualClinic; Anxiety disorder clinic sample were outpatients of the Clinical Research Unit for Anxiety and Depression; National Survey sample were from the 2007 Australian National	Anxiety and depression (T)	iCBT VirtualClinic programs

	Mental Health Survey		
[49] Internet survey sample were aged 18 years or over, 29% male, 51% single/never married, 47% with diploma/degree or above education, 64% employed<br>(SEX, EDU, SE)	Age >=18, with obsessive compulsive disorder | Internet clinic sample recruited from open survey on VirtualClinic; Anxiety disorder clinic sample were outpatients of the Clinical Research Unit for Anxiety and Depression; National Survey sample were from the 2007 Australian National Mental Health Survey	OCD (T)	Hypothetical Internet therapy, defined as treatment delivered via the internet
[50] Older adults with anxiety; mean age 66 years (SD = 4.6), 68% female, 59% married/defacto, 32% employed, 50% with tertiary education, 55% reported previous mental health treatment, 36% spend >10 hours per week on internet<br>(SEX, EDU, SE)	Australian residence; age >=60; access to computer Internet, and printer; at least mild anxiety as a mental disorder; not participating in CBT; not using illicit drugs or consuming >3 standard drinks/day; not schizophrenia or bi-polar; not severe depression; not taking benzodiazepine | Sourced online	Anxiety (T)	Managing Stress and Anxiety program; iCBT for older adults with anxiety; 5 online lessons; 8 weeks; clinical psychologist moderated online discussion forum; email contact with clinician; automatic reminder and notification emails; information resources

CBT= cognitive behavioural therapy; GAD = Generalised anxiety disorder; iCBT=internet-based cognitive behavioural therapy; iIPT = internet-based interpersonal therapy; PD = panic disorder; SAD = social anxiety disorder; RCT = randomised control trial.
Mental health disorder targeted: P = Prevention; T = Treatment
Categories of characteristics referred to in text: EDU = education; YP = young people ; 60+ = people over the age of 60; SE = socio-economic status; U-R = geographic distribution (e.g. urban versus rural residence); ETH = ethnicity
