# Supplementary material for: Establishing and Governing e-Mental Health Care in Australia: A Systematic Review of Challenges and A Call For Policy-Focussed Research
Source: J Med Internet Res. 2016 Jan 13;18(1):e10. doi: 10.2196/jmir.4827 (PMC4730106; doi:10.2196/jmir.4827)
Supplement: Supplementary file 3 [file jmir_v18i1e10_app3.pdf]

Multimedia Appendix 3. Measurements related to facilitating uptake and evidence on e-mental health utilisation (N = 17).

**Measurements related to facilitating uptake**	**Results on e-mental health utilisation**
[23] Mode of service: face-to-face, over the phone, help online, and no help	Face-to-face help was preferred by 58.9% of participants, 23.8% of the sample preferred to not seek help, 16% preferred online help and 1.3% stated that they would prefer to seek help over the phone.
[24] Presentation of method of e-mental health information: text, film, and control (no information); Type of service: information websites, online counselling, online program with therapist assistance, and online program without therapist assistance	Participants presented with information about e-mental health by text reported higher likelihood of e-mental health use in the future than participants not presented with information, whereas there were no differences in likelihood for participants presented the information by film; Participants perceived online programs without therapist assistance as being significantly less helpful, and reported reduced likelihood of engaging in these programs in the future when compared to other e-mental health services.
[26] Factors discouraging and encouraging participation	The most common factors that discouraged participation were too busy (>40%), just not interested (>20%), and prefer to deal alone (>10%). The most common factors that encouraged participation were don't know/nothing (>30%), financial incentive (>20%), more free time (>15%)
[27] Adherence, attrition, treatment satisfaction	High adherence and satisfaction, including likelihood of recommendation; 80% completion rate
[28] Treatment satisfaction	Within participants, 90% and 78% indicated that they were very satisfied or mostly satisfied with the course, 100% and 92% indicated that it was worth their time, and 95% and 100% indicated that they would recommend the course to a friend for the Anxiety Trial and Depression Trial, respectively.
[29] Feasibility and acceptability	Qualitative study
[31] Preferences for different modes of e-mental health: accessing a website for information; website with a question and	The most preferred modes of e-mental health were website with information (preferred by 48%-62% males, 60%-70% females), online

answer service that sends SMS or emails; online clinic; interactive single-player games teaching life skills; interactive multiplayer games teaching life skills; males less likely than females to talk about their problems online	clinics (M = 39%-52%, F = 49%-56%), and website with question and answer service (M = 29%-42%, F = 42%-49%)
[32] Gender-identity (male), age (16-24), open-ended (qualitative) exploration of variables influencing help-seeking.	Strongest preferences among e-mental health options were to use websites with information and/or fact sheets (48%), website with online clinics (40%), website with information and multimedia content (30%), and website with question and answer service that sends SMSs or emails (30%).
[33] Acceptability by severity of symptoms; preferences between face-to-face or internet treatment	The majority of both lay participants and health professionals, respectively, stated a preference for face-to-face therapy (58% and 71%), followed by no preference (33% and 25%), with only 9% and 4% choosing internet; health professionals were less likely to choose internet; strongest barriers for uptake was a lack of information and knowledge around e-mental health
[34] Treatment acceptability and satisfaction at post-treatment	85%-82% rated the course and lessons as very satisfied or mostly satisfied; 100% reported ‘it was worth their time doing the course’ and that they ‘would recommend the course to a friend’
[35] Age, gender, region of residence	Middle-aged rural females were most likely to have used e-mental health information in the past 12 months (18.1%); older rural males were least likely to use (2.2%)
[36] Reduction in symptoms	100% satisfaction. Similar improvements in symptoms to clinical trials.
[42] Demographic and mental health factors associated with adherence	Adherence rates were higher in school-based settings (45% completed 3+ modules) compared to community settings (<10% completed 3+ more modules).
[45] Demographic and mental health	76% reported an interest in using a mobile phone based e-mental health program
[47] Open-ended (qualitative) exploration of variables influencing likelihood of practitioner referral to online mental health resources	Qualitative study of rural practitioners
[49] Age, symptom severity, education, sex, marital status, employment	82% believed Internet therapy would be useful. 98% would be willing to try Internet therapy.<br><br>Key perceived advantages reported included: reduce time, cost, and travel (>60%), privacy and anonymity (56%), reduce embarrassment (33%),

	symptoms not severe enough for face-to-face (14%), and treatment not available where they live (11%).<br><br>Key disadvantages reported included: prefer face-to-face (10%), need to see the person they are talking to or community concerns (8%); do not understand what e-mental health is (6%), and prefer self-help (3%).
[50] Treatment satisfaction	Participants reported a high level of satisfaction with the overall program, with 100% of the participants reporting that they would recommend the program to a friend. All participants also reported that it was worth their time doing the program.
